# Economic and reliability assessment of intermediate charging ports for EV stations

**DOI:** 10.1038/s41598-025-26362-w

**Published:** 2025-11-27

**Authors:** K. Vaishali, D. Rama Prabha

**Affiliations:** School of Electrical Engineering, Vellore Institute of Technology (VIT), Vellore, Tamil Nadu 632014 India

**Keywords:** Charging port configurations, Electric vehicles, Failure rate evaluation, Reliability probability of charging port, Voltage stability of charging station, Distribution system, Energy science and technology, Engineering

## Abstract

The widespread adoption of electric vehicles (EVs) poses significant challenges to distribution system operations. When electric vehicle charging stations (EVCS) are installed without proper planning, they can negatively impact voltage stability and reliability in distribution systems leading to reduced customer satisfaction. To enhance distribution system charging station reliability, a novel 36-port design has been developed, incorporating identical and non-identical port configurations. This system operates within a 50–350 kW distribution network. The research employs failure rate analysis based on MILHDBK217F standards, while port probability functions for failure rate and reliability are assessed according to MILHBK-338B guidelines. The study introduces an evaluation process to determine charging station success rates based on individual port failure rates. The failure rate analysis for the charging station with 36-port configuration utilizes binomial distribution method. Additionally, the research includes a price assessment framework for the 36-port system, considering both failure rates and individual port maintenance success rates. The study evaluates distribution station voltage stability by examining individual port failure rates and the overall reliability of the 36-port configuration. Results demonstrate that the proposed design achieves reduced failure rates and maintenance costs while maintaining superior port arrangement reliability and voltage stability.

## Introduction

Climate change and global warming concerns have recently sparked increased interest in plug-in electric vehicles (PEVs) over conventional fuel-powered vehicles. The rising demand for electric automobiles is primarily driven by two factors: pollution concerns and fossil fuel scarcity. Governments are now offering financial incentives to companies that establish EV charging infrastructure. The growing demand for charging EVs poses significant difficulties facing power system engineers. EVCS require strategic distribution in densely populated areas with high charging demands, necessitating careful planning. It’s essential to minimize power losses while maintaining power grid voltage variation within acceptable regulatory limits. Additionally, the cost of land in proximity to potential charging station locations is a significant consideration. The strategic planning of charging station placement is further complicated by various uncertainties associated with electric vehicles.

### Background and motivation

We describe the rapid uptake of EVs, key challenges including voltage rise, distribution loss and charging infrastructure. This motivates the design of charging stations that trade-off between reliability, cost and voltage stability.

The research in^1^ presents a comprehensive methodology for evaluating how different levels of PEV adoption impact distribution network investments and incremental energy losses. This approach employs an advanced large-scale distribution planning model, applied to two real-world distribution areas. The study reveals that distribution network investment costs could increase by up to 15% of total actual investment expenditures. Moreover, during off-peak periods, energy losses might rise by as much as 40% in scenarios where PEVs constitute 60% of the vehicle fleet. Examining the effects of extensive Plug-in Hybrid Electric Vehicle (PHEV) powering on lines is essential to account for their great numbers and complicated charging patterns. Many unknown factors influence the charging behaviour of PHEVs, particularly local dissemination or regional transmission networks, hence generating general demand for charging uncertainty. In^2^, probabilistic power flow (PPF) analysis offers a method to evaluate PHEV charging impacts on power grids under these uncertain conditions. The approach introduced in this paper develops a unique model for forecasting PHEV charging requirements, then applies queuing theory to explain the behavioral dynamics observed across multiple PHEVs.

### Identified research gaps

Most of the previous works (e.g.,^3,6,10^) focus only on the optimal locations/capacities of EVCS with no port-level reliability analysis. Most of existing works minimise the economic or power loss, however, little attention has been paid to failure rate analysis for charging ports to evaluate their long-term operational reliability. However, few comparisons are available between identical and non-identical port configuration that have pressing relevance for installation and maintenance.

In this paper, the multi-objective optimization problem of finding the location and the size of parking facilities operating as vehicle-to-grid (V2G) power sources in distribution systems as a new type of distributed generation (DG)^3^ is formulated. System reliability, power loss, and capital cost are considered in the approach. The study identifies a critical research gap in system stability analysis, specifically the lack of exact load models accurately defining EV loads. To address the present gap, study creates a static load model that provides an essential basis for realistic stability analysis assessments^4^. The results of the research show that including EV fast-charging stations can have a significant impact on the power system’s stable voltage.

## Research objectives

To suggest and investigate a new 36-port EV charging station layout with mixed same-sector and non-same sector port structures. To estimate failure rate and reliability probability by MIL-HDBK-217F and MIL-HDBK-338B approaches. An evaluation of the maintenance costs required on different port geometries. For voltage stability performance through the IEEE 13-bus test system.

Unregulated charging and discharging of electric vehicles poses potential risks to power system security and reliability, necessitating intelligent scheduling solutions. This study proposes a methodology for smart management and scheduling of large-scale EV fleets within urban parking structures^5^. The parking facility’s energy management system successfully balances both economic and technological objectives. Additionally, the system creates income-generating opportunities for EV owners through vehicle discharging while ensuring their specified state of charge (SOC) requirements are met by departure time. The research in^6^ employs probabilistic methodologies to model EV behavior patterns. It also introduces an approach for strategically positioning EV parking facilities with reliability constraints as a primary consideration.

Research in^7,8^ identifies PEV parking lots as a key solution to charging station requirements. This paper addresses distributing system losses to reduce total system costs, including those connected to power loss, network dependability, and voltage fluctuation. From an economic point of view, the system not only increases losses in the distribution network but moreover improves parking lot ease of access. Research intends to determine the optimal number, location, and availability of EV parking spaces to optimize earnings for the distribution of electricity companies. Furthermore, the projected rate at which EVs will grow in the following years is treated as a probabilistic parameter^9^. Studies^10^ present numerical results demonstrating how the proposed optimization approach achieves minimum costs for charging station placement while simultaneously ensuring reliable charging and maintaining preferred service excellence. This is accomplished through analysis of electric vehicle owner and driver behavior patterns.

Evaluating the advantages of power exchange among parking facilities, distribution networks, and EVs requires establishing an optimal schedule and determining appropriate timeframes for EV charging and discharging operations. To meet this need, an innovative method for EV charging scheduling is presented^11^. Improper charging station installations have adverse effects on energy distribution systems, which presents a major difficulty. This has to do with the best strategic choices procedure for sizing and positioning charging system in cities. Studies show enhanced voltage profile and quality using genetic algorithms and particle swarm optimization methods^12^. Studies introduce a two-phase approach. The first phase evaluates the capacity of existing distribution network infrastructure to satisfy PEV charging demands. The second phase aligns public PEV demand expansion with installed fuel cell system (FCS) capacity using a staging plan model that is economically optimum^13^.

### Key contributions/deliverables

Organized procedure for port level reliability evaluation of EVCS based on binomial probability distribution. Comparison of identical and non-identical configurations with respect to reliability, cost and stability. A detailed case study showing that the 36-port design performs with less failures and maintenance cost, better voltage profile as compared against existing strategies.

The need for EVs has increased dramatically worldwide due to its environmental and economic benefits. However, integrating large numbers of electric vehicles into distribution systems creates challenges, including voltage volatility, increased upkeep expenses, and less dependability of charging systems^14^. These issues are critically important within distribution systems and require dedicated attentiveness. The uncontrolled running of EV charging stations presents significant distribution obstacles for networks. The random charging patterns caused by EV fleet usage create multiple difficulties for the charging infrastructure. Charging station deployment depends on several factors, including geographic location, local EV user density, and investor financial conditions. The evaluation of parking lot charging port configurations considers factors such as total land area and distribution system power supply capacity. Achieving optimal power load distribution across the system remains a critical priority^15,16^.

Voltage stability has become a worldwide issue of increasing importance as economic and environmental constraints cause most power systems to run close to their security limits. Current research on EVCS planning significantly lacks consideration for power system uncertainties, including demand variations and impacts from electric vehicle integration^17,18^. This makes deterministic power flow (DPF) approaches unreliable for EVCS planning. To deal with this issue, we proposed a stochastic load model for EV charging and introduced a novel metric, namely dynamic system voltage stability (DSVS) in^19,20^. We tested our proposed method in a case study based on the IEEE 13-node planning area map and a distribution network structure diagram.

The IEEE 9-bus radial distribution system served as the test network for this study. Analysis was conducted across four possibilities using both predictable and uncertain methods at the same time. Results revealed that charging strategies significantly impact systems for charging EVs, especially in addressing integration challenges of EV chargers^21,22^. Charging stations are essential for vehicle battery charging. The increased power demand from EV charging causes distribution systems to experience greater power loss and voltage drops. Strategic placement of charging stations at optimal nodes is critical for maintaining healthy voltage profiles and reducing power loss. This article details the arrangement of electric vehicle charging stations across the IEEE 13-node radial system^23,24^. Based on these considerations, the following section evaluates charging station reliability according to port configurations. The novelty of this work is highlighted in Table 1 below.

**Table 1:** Key novel features and contributions of the proposed work.

Novel Feature	Description / Advantage
Hybrid 36-port layout (identical + non-identical ports)	Combines best of homogeneous and heterogeneous port configurations in the same station for easy scalability without sacrificing availability
Component-level reliability modeling using MIL-HDBK 217F / 338B	Failure rates estimated at the piece-part level (power electronics, converters, connectors and control modules) provide a finer-grained station reliability input
Coupled maintenance cost modeling per port	Maintenance cost is linked to failure rate, enabling economic assessment of reliability interventions (e.g., redundancy, spares)
Integration with voltage stability index (VSI) analysis	Port-level failures are mapped onto grid-level voltage behavior (IEEE-13 bus), enabling connected stability assessment
Per-unit normalization bases clearly defined	The base cost and reliability value (for per-unit based values) for the metrics are clear and consistently applied across all results
Comparative evaluation across configurations	We compare multiple configurations (e.g. different mixes of identical/non-identical ports) under the same reliability/cost / stability framework to show performance trade-offs

## Methodology

**Fig. 1:**
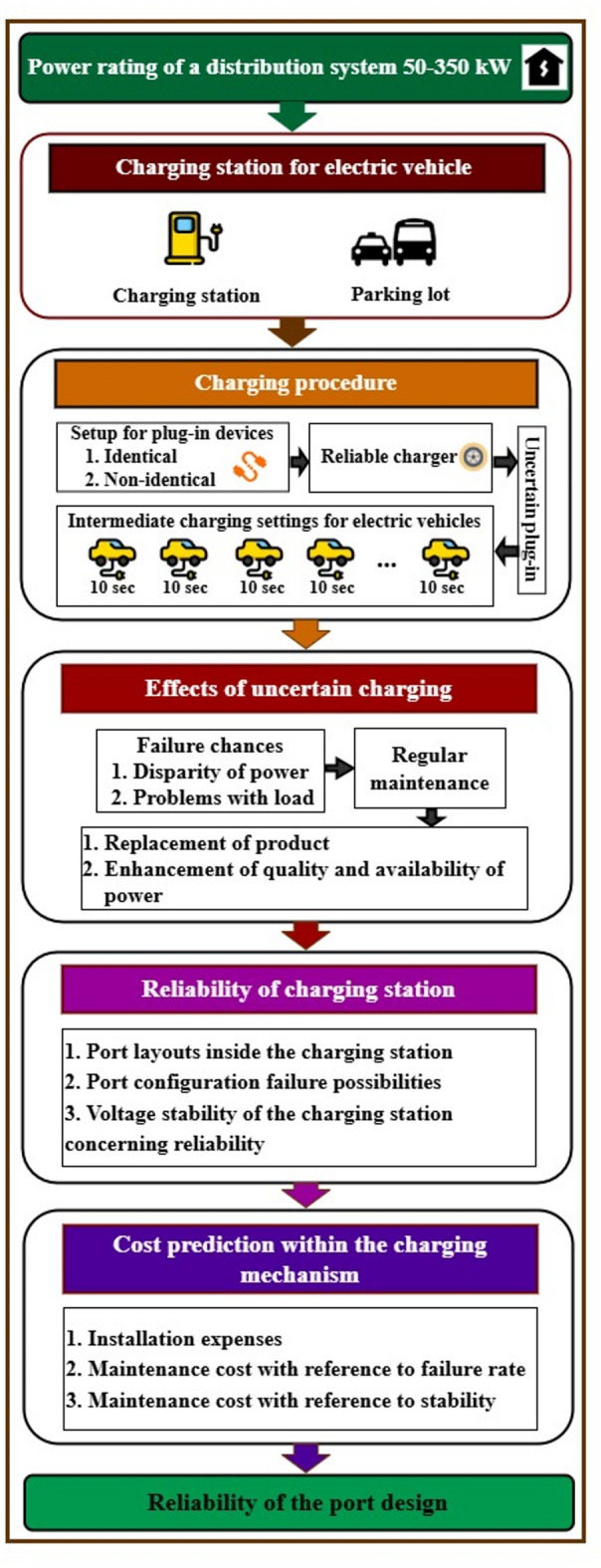
The suggested assessment approach for charging port dependability in terms of failure rate.

Available car parking space mostly decides the installation capacity of the charging station^25^. But, setting up and connecting charging ports depends on more than just parking availability. Capital investment and expenses needed to maximize port capacity in a given area drive most of the installation process. To correctly allocate cash for purchase, the chosen port configuration should be subject to reliability testing before installation. Based on these considerations, an approach for estimating dependability has been presented, as illustrated in Fig. 1.

The first step in determining charging port arrangement reliability involves considering uncertain plug-in conditions^26,27^. Both identical and non-identical port designs have been evaluated to accommodate uncertain plug-in systems, as shown in Fig. 1. Fluctuating loads on ports under EVs’ varied operating conditions can cause greater failure rates at charging stations. Failures of charging stations are connected to different power rating needs of consumers. In order to provide charging continuously, it is necessary to replace failed ports since it impedes the operations by a frequent port failure. If the port fails, which is a function of material quality, it shall be replaced and be consistent with capital cost requirements for port maintenance. Cost estimating has been included to allocate maintenance dollars throughout the purchase of superior replacement parts for charging system improvement. The product lifetime has been simulated and validated in order to ensure the quality according to the MIL-HDBK- 217F military handbook criteria. According to MIL-HDBK217F military handbook criteria, product lifetime has been assessed to confirm quality^28^. Following the suggested approach, dependability has been calculated as a rate of failure for each charge port setup. Port design and configuration determine the ability as well as capabilities regarding the charging station. Although same port designs are typical for EV charging stations, their installation calls for extra room.

Giving equal parking spots for car charging is somewhat difficult. Because non-identical structures have to fit different power capabilities, identical ports provide benefits in terms of simpler diagnosis and reduced maintenance expenses over non-identical ports. While non-identical port configurations work better in limited installation areas, identical ports excel in reduced maintenance, extended lifespan, and operational reliability. Based on these considerations, both identical and non-identical preparations for ports have been analyzed for their installation characteristics, failure rates, reliability, and maintenance costs. Based on this study, a 36-port charging station with both identical and non-identical port layouts has been suggested. The next section outlines the layout and design of the ports in the suggested 36-port charging station, and the comparative review table is given below in Table 2.

**Table 2:** Comparative review of the 36-port reliability framework with recent EVCS and V2G planning studies.

Study (Year)/Citation	Scope/Focus	Methods (reliability/planning)	Reliability metrics/base	Test data/system	Key findings (short)	How the current 36-port work differs
Powell et al., NREL report^36-39^, (2025)	Relationship between station reliability, station resilience, grid resilience, and EV adoption	Data analysis + literature review; empirical charger-level availability metrics; scenario analysis	Availability / uptime metrics (empirical % uptime) — industry-level baselines and effects on adoption	Large real-world datasets and aggregated station data	Station reliability critically affects adoption and grid resilience; real uptime is often lower than provider claims	Your work complements this empirical perspective by providing a component-level failure-rate/reliability model (MIL-HDBK-based) that can inform station uptime estimates used in such adoption studies
Pilotti et al. (2023)-optimal e-fleet station design with V2G and M.wang et al.^40-43^, (2024, 2025)	Planning and optimization of charging stations with V2G capabilities	Bi-objective optimization (capacity & scheduling), techno-economic modelling	Reliability often implicit (service constraints), not MIL-HDBK explicit	Simulated station models / optimization scenarios	Demonstrated benefits of V2G-enabled designs for cost & service, but did not model detailed port-level failure dynamics	Your study adds micro-level reliability (failure rates & maintenance cost coupling) that can improve the realism of V2G planning models by accounting for port failures and maintenance costs
Niu et al. (2024) — V2G-enabled charger deployment and M. Nour^44^	Optimization of V2G-enabled charger siting & operations	Mixed-integer optimization with operational constraints, scenario analysis	Reliability handled via operational availability constraints (probabilistic scenarios)	Modeled networks with demand scenarios	Optimizes placement and operations under V2G; reliability/tolerance modeled at high level rather than component life models	Your 36-port paper supplies component failure models (MIL-HDBK based) and per-port maintenance cost calculations that can be used to refine the operational availability assumptions in such planning studies
Karunarathna et al. (2024) — Reliability analysis of fast charging systems and M. S. Saha et al.^45^	Reliability of fast chargers; applicability of MIL-HDBK-217F for power electronics	Reliability prediction using MIL-HDBK-217F; fault-tree / statistical analysis; life prediction	Failure rate λ (per million hours) using MIL-HDBK methods; component-level λ	Subsystem / component models for fast chargers	Confirms MIL-HDBK methods are useful for predicting lifetime of power electronics in chargers and recommends component-level modelling	Your framework applies the same MIL-HDBK approach but at station layout scale (36 ports) with identical vs non-identical batch analysis and with direct coupling to per-port maintenance cost and VSI (voltage stability index) analysis
ESIG / Grid planning: “Charging Ahead” (2024) — industry best practices and R. Brahmachary et al.^46^	Grid planning & good/better/best practices for vehicle electrification	Practitioner guidance, planning workflows, stakeholder roles	Not a MIL standard-uses planning KPIs, availability, hosting capacity	Industry case studies / planning exercises	Emphasizes need for multi-stakeholder planning & reliability-aware grid studies to support EV rollout	Your work bridges the planning/practitioner gap by providing a quantitative reliability + cost metric per port that planners can plug into grid-level assessments (e.g., maintenance budgets, expected downtime scenarios)
ChargerHelp / Charger reliability assessments & industry analyses (2023–2024) — empirical studies / press summaries and R. Brahmachary et al.^47^	Empirical evidence of real-world charger downtime and user experience	Large empirical audits and field data; user reports/field inspections	Uptime / downtime percentages, service failures (empirical)	Thousands of public chargers (real world)	Many public chargers have lower uptime than reported; identifies ghost/zombie stations and systemic maintenance issues	While empirical work reports realized station uptime, your 36-port model estimates underlying causes (component failure rates and maintenance cost) that can explain and predict such empirical uptime values and help design stations with higher operational availability
Vaishali & Prabha (2024) — Reliability & Economic Evaluation approaches ^26^	Reliability evaluation methodologies for various port configurations	MIL-HDBK based failure-rate estimation; simulation / binomial distributions	Failure rate per 10^6 h (MIL-HDBK normalization), cost functions	Simulated station configurations	Presents an evaluation approach for port reliability and some comparative results for configurations	The present 36-port manuscript is an extension/application: it combines identical & non-identical ports in a single 36-port CINPS design, links port reliability to VSI on IEEE-13 bus, and provides explicit per-unit maintenance cost bases (clarified per reviewer)

A. The suggested port construction layout of a combinational logistics identical and non-identical setting up.

**Fig. 2:**
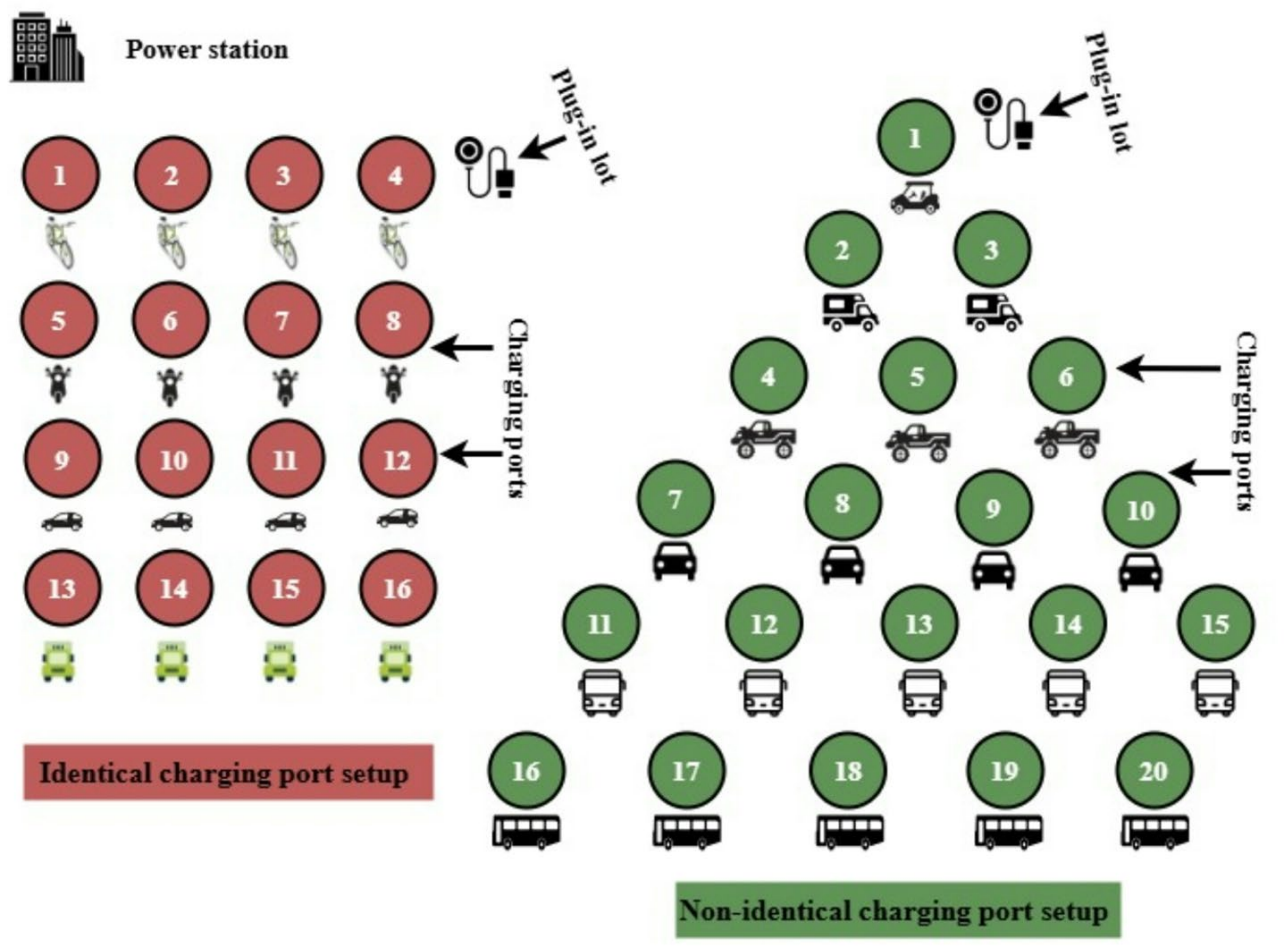
Suggested 36-ported charging station setup (**a**) 16-ported identical setup; 20-ported non-identical setup.

The present paper proposes a 36-port charging station using a combination of identical and non-identical port systems (CINPS) arrangement^29^. Design prioritizes ease of vehicle connection while parked, reliability, and reduced maintenance expenses. The structure features 16 identical ports and 20 non-identical ports, for a total of 36 ports. Figure 2 provides a visual representation of this proposed charging station configuration with its mixed identical and non-identical port design.

**Fig. 3:**
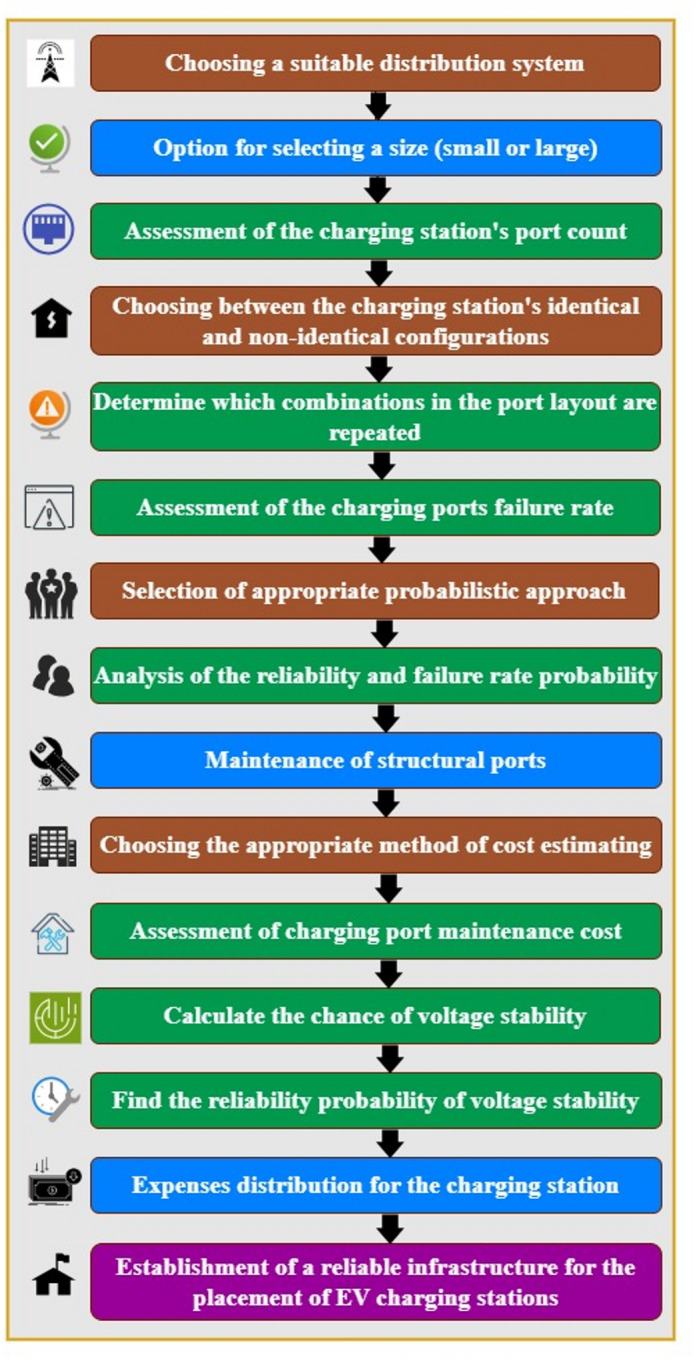
Evaluation process for the reliability of charging ports in the charging station.

The suggested 36-port charging station, with its various port layouts, is shown in Fig. 2. Figure 2a illustrates the 16-ported identical configuration, arranged in batches of 4 ports each. Figure 2b displays the 20-ported non-identical configuration with unequal port arrangements across individual batches. Both identical and non-identical port layouts combined produce the whole structure depicted in Fig. 2. To investigate the performance of the identical and non-identical systems, failure rate, reliability and cost function are analysed. The intended 36-port charging station operates at power ratings of 50–350 kW to maintain voltage and current values necessary. By using value of rate of failure estimation method illustrated in Fig. 3, the first measurement in testing the reliability of the charging station has been tested.

B. Methodology for assessing EV charging station port dependability.

This study offers a gradual assessment method depicted in Fig. 3 to evaluate many charging port configurations in a 36-port charging station. Reliability criteria directed the distribution system selection procedure^30^. Binomial distribution was chosen to evaluate the necessary capacity for the 36-port station, which features both identical and non-identical configurations. The charging station’s effectiveness depends on well-designed port patterns compatible with parking lot capabilities. Port reliability, measured by failure rate, determines charging facility quality. Since port failures may occur during intermittent EV charging, both real and theoretical statistical validation methods are needed to assess reliability.

The research uses binomial distribution to analyze probability functions of the 36-port configuration because it can simultaneously evaluate and compare two different probability sets. This methodology helps determine reliability in terms of failure rates for both identical and non-identical port configurations. A charging station with 50–350 kW power capacity was selected for this study. The analysis followed standard workflows for identical and non-identical systems. The setup includes 36 ports with added reliability assessment methods. Random EV charging processes were considered when evaluating reliability across different combinations. The parking facility features a dual-batch charging port system. The identical configuration contains 16 individual ports (2 vulnerable) while the non-identical system has 20 ports, with repeatability methods used to determine product reliability. Repeatability occurs whenever random arrangements are selected. In^31^, the system interface failure rate was evaluated using MILHBK-338B standards and reliability analysis was conducted using Eqs. (1) to (8).

### Problem statement

Aforementioned assessment method has been used to determine the dependability likelihood of charging ports connected to the charging station. Following Eq. (1) to (8), the failure rate and dependability have been assessed. The projected failure rate is computed using the MIL-HDBK-217F model^28^, as shown in Eq. (1)-(5),

Projected failure rate (λ_E_)

1 $$\lambda_{E} = \frac{{\lambda_{physical} }}{Total unit operating time \times Annual period}$$

*where *$$\lambda_{E} = \, Environmental \, failure \, rate$$

$$\lambda_{p} \, = \, \lambda_{b} \pi_{{\text{T}}} \pi_{{\text{R}}} \pi_{{\text{S}}} \pi_{{\text{A}}} \pi_{{\text{Q}}} \pi_{{\text{E}}} \;Failure/10\hbox{-}^{6} Hours$$

$$\pi_{T}$$ *: Temperature component.*

$\pi_{R}$ *: Present rating element.*

$\pi_{S}$ *: Stress factor for voltage.*

$${\uppi }_{{\text{A}}}$$ *: Application element.*

$${\uppi }_{{\text{Q}}}$$ *: Quality feature.*

$$\pi_{E}$$ *: Environmental element.*

Electrical component lifetime is greatly influenced by the temperature. The temperature element relies on the component’s applied current and voltage. This shows the temperature component.

2 $$\pi_{T} = \exp \left( { - 3082\left( {\frac{1}{{T_{j} + 273}}} \right. - \left. \frac{1}{298} \right)} \right)$$

*where Tj ambient temperature.*

0ºC is equal to the 273º K.

23ºC is regarded as the room temperature.

Choosing components calls for careful thought on both application characteristics and environmental factors. The constant value of the environmental component relies on the application of the product. Let us examine the charge application (Ns).

*Environmental element (*$\pi_{E}$*)* = *N*_*S*_ = *fixed value.*

The kind of material utilized in its manufacture decides the quality and lifetime of the product, such as JANTXV, JANTX, or JAN. the rate of component failure is directly impacted by quality, making it a significant determinant of product quality. It is represented by the symbol of $\pi_{Q}$.

3 $$\small {\text{Quality factor }}(\pi_{Q}) \, = {\text{ material }} = {\text{ constant}}$$

The voltage delivered to power devices plays a crucial role in determining the product’s failure rate during operation. High failure rates can occur due to excessive blocking voltages and peak currents, with voltage stress and current ratings being the key contributing factors.

4 $${\text{Present rating element }}(\pi_{R}) \, = \, \left( {I_{rms} } \right)^{0.40}$$

*where I*_*rms*_ = *present port rating*

5 $${\text{Stress factor for voltage}} = \, \left( {V_{s} } \right)^{1.9}$$

*where V*_*s*_ = *stress from voltage*

The real system failure rate follows the conventional reliability definition from MIL-HDBK-338B^31^ and the binomial probability of system success is given by Eq. (7) ^22^,

Real system failure rate (λ)

6 $$Failure rate (\lambda ) \, = \frac{Number \, of \, failures}{{Total \, operating \, time \, of \, units}}$$

Probability of the system’s succeeding P(s)

7 $$P(s) = \sum\limits_{i = x}^{n} {\frac{n!}{{X!(n - X)!}}p^{X} . } q^{n - X}$$

*where n* = *No. of plugsr = No. of permitted channel failuresp = Probability of each channel’s successq = (1-p)q = probability of each channel’s failureX = The quantity of successful channels*

The unknown element (Uλ) is adapted from reliability prediction guidelines in MIL-HDBK-217F^28^ and reliability design handbooks^31^,

Unknown element (U_λ_)

8 $${\text{U}}_{\lambda } = \frac{1}{4r}\left( {Z_{0.95} + \sqrt {4r + 3^{2} } } \right)$$

**Fig. 4:**
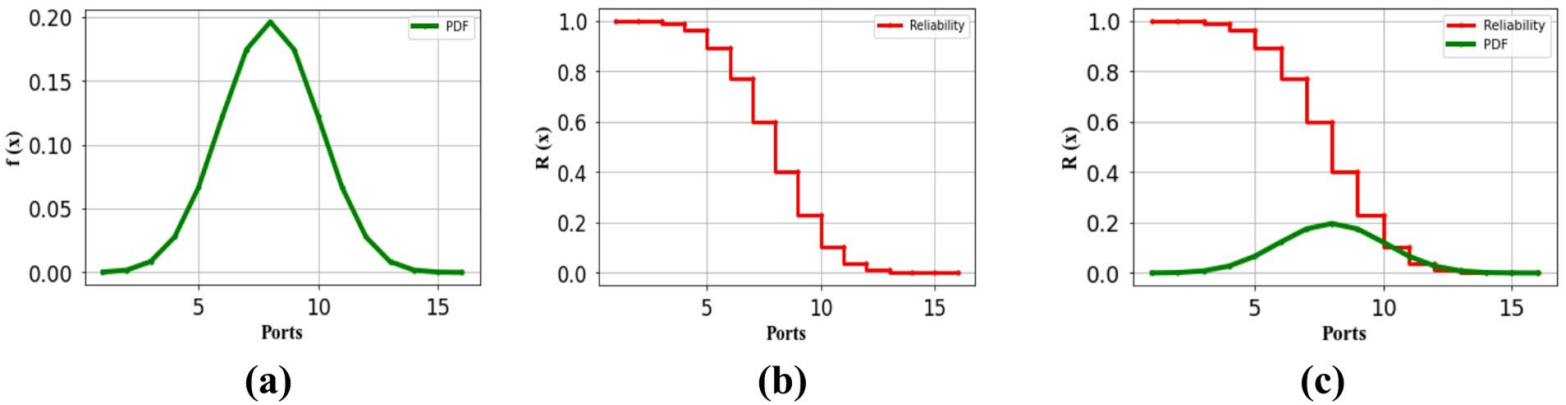
(**a**) Failure Probability of the 16-ported identical batch charging oriented parking lot for EV (**b**) Reliability of the 16-ported identical batch charging oriented parking lot for EV (**c**) Cumulative comparison of reliability of the system as per the failure rate.

The reliability of the charging station has been analyzed using standard reliability assessment techniques, based on a combinational batch charging setup with up to 36 ports. Figure 4 presents the system’s reliability in terms of its failure rate.

### The conversation over the suggested 36-port configuration’s simulation findings

The suggested 36-port EV charging station’s dependability evaluation began following the established evaluation methodology. The station’s design incorporates a combined structure of 16 identical and 20 non-identical charging ports. In accordance with standard evaluation procedures, failure rates were calculated by considering potential repetitive patterns within both port configurations in the charging area. Table 3 presents the comprehensive results of the failure rate and reliability probability analysis for both the 16-ported identical and 20-ported non-identical charging configurations.

**Table 3:** Combination of identical and non-identical oriented charging station porting failure rate and dependability.

Charging port count (n)	Port’s batch order arrangement	Anticipated failure rate	System failure rate (q)	System success probability P(s)
36	16 (Identical)	2%	8%	0.5
	20 (Non-identical)	2%	2%	0.5

Table 3 clearly shows the rate of failure analysis for the 36-port configuration, divided into 16-ported identical and 20-ported non-identical structures. While both configurations share equal expected failure rates, their repetitive possibilities differ based on port structure. The data demonstrates that the identical 16-port system effectively supports EV charging with a 2% failure rate and reliability of **0.5 per 10^6 h**. Meanwhile, the non-identical 20-port configuration operates with a failure rate of 2%. The analysis reveals that the performance with a **0.5 per 10^6 h** success rate for both the identical and non-identical configurations. Nevertheless, the identical system’s **0.5 per 10^6 h** success rate remains acceptable for high-capacity transportation EVs, despite its maximum 8% failure rate. In conclusion, this 36-port charging station achieves port activities with only a 2% failure rate, showing a better success rate. Figure 4 in the following section provides a visual representation of both the 16-port and 20-port configurations’ failure rates and reliability metrics.

A. The assessment of the suggested charging port arrangement on the reliability of each individual port.

The performance evaluation of each port in the 36-port system was conducted using various repetitive combinations (Rc) based on whether the configuration was identical or non-identical. The system design incorporates two distinct port arrangements: a 16-ported identical configuration and a 20-ported non-identical configuration. Initially, the failure rate assessment began with the 16-ported identical configuration. All simulation analyses were performed using python in the anaconda software environment. For the 16-ported identical configuration, the charging station failure probability calculations incorporated a two-port combo. Based on these repeated combinations, Fig. 4 offers a thorough visual depiction of the failure rate, dependability, and cumulative comparison of the system.

**Fig. 5:**
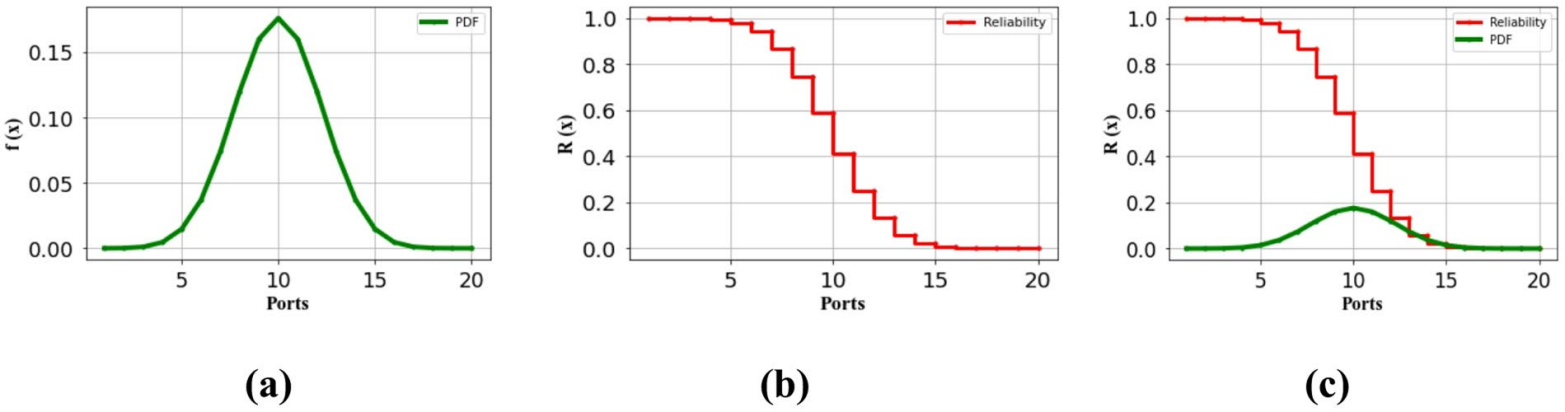
(**a**) Failure Probability of the 20 ported non-identical batch charging oriented parking lot for EV (**b**) Reliability of the 20 ported non-identical batch charging oriented parking lot for EV (**c**) Cumulative comparison of reliability of the system as per the failure rate.

The 16-ported identical configuration features a systematically arranged 4-port batch design to minimize installation space. As shown in Fig. 4, this setup gives every port a low failure rate of 0.20 per 10^6 h. System performance was assessed through reliability measurements based on individual port failure rates, as reliability directly correlates with product lifecycle. The reliability of each port configuration was assessed throughout time to standardize the statistical analysis, as depicted in Fig. 4a and b. Reliability R(x) was computed over time based on individual port failure rates and visually compared across ports in Fig. 4c. Figure 4a illustrates that although the failure rates for each charging activation are negligible, the concurrent use of chargers markedly elevates the failure rate of the port configuration. Figure 4b clearly demonstrates how individual port failures impact overall system reliability. Figure 4c offers a visual depiction to elucidate the correlation between failure rate and dependability. This graphic verifies that the 16-ported identical design can endure prolonged charging durations with a dependability of 0.20 per 10^6 h and a failure rate of 0.14 per 10^6 h. Figure 5 illustrates the performance assessment of the secondary charging station arrangement.

Within the 36-port system, 20 ports are arranged in a non-identical configuration for EV charging. The failure rate and reliability assessments for each particular port in this 20-ported non-identical arrangement are presented in Fig. 5. This configuration features port batches structured with different repetitive combinations of ports. Figure 5a, b, and c illustrate the failure rate and reliability metrics for a 2-port repetitive combination in the 20-port system. With a peak failure rate of 0.18 per 10^6 h, Fig. 5a shows the 20-port charging system can provide constant charging. Port arrangement’s repeating combination causes the system to first display a greater failure rate of 0.16 per 10^6 h. The 20-ported non-identical arrangement offers EV charging with a dependability of 1 per 10^6 h based on this failure profile. When employing a repeating combination, Fig. 5c verifies that this setup stays operational with a 0.18 per 10^6 h failure rate and 1 per 10^6 h reliability.

Ultimately, the 16-port similar architecture in the 36-port system offers better charging capabilities with a dependability of 0.20 per 10^6 h as compared to the 20-ported non-identical setup. Table 4 presents the success rates for each individual batch within the 36-port charging station’s identical and non-identical arrangements.

**Table 4:** Contrast with the success percentage of charging ports working in a 36-port batched arrangement (identical and non-identical).

A combination of the batch system with 36 ports	Batches	No.of Ports/Batch	Healthy Ports	Success Rate
4	16-ported batch system			
	B-1	4	0.005	6.25
	B-2	4	0.005	6.25
	B-3	4	0.005	6.25
	B-4	4	0.005	6.25
	20-ported batch system			
6	B-1	1	0.008	0.008
	B-2	2	0.013	0.0256
	B-3	3	0.0213	9.663
	B-4	4	0.035	1.5006
	B-5	5	0.004	6.3489
	B-6	5	0.004	6.3489

Table 4 reveals according to the planned 36-port charging station accommodates a maximum of 4 ports per batch in the identical arrangement. The non-identical configuration can incorporate up to 5 switches in a single batch and as few as 1 port in another, based on possible 2-port repetitive combinations. The 16-ported identical categorization maintains a consistent port success rate of 6.25 per 10^6 h across all batches due to equal distribution of functional ports. In contrast, the 20-ported non-identical configuration features varied port structures, resulting in success rates ranging from 0.008 to 6.3489 per 10^6 h for 2-port repeating pairings, as indicated in Table 4. The data confirms that the identical 16-port structure represents the superior configuration within the 36-port charging station. Although the non-identical system gives acceptable success rates, it requires regular maintenance to ensure consistency, leading to higher maintenance costs. Product maintenance costs correlate directly with quality and reliability. While highly reliable products reduce maintenance needs, they require greater initial investment. Consequently, maintenance cost estimates have been developed for the proposed 36-port charging station configuration. According to^32^, the essential parameters for calculating maintenance costs will be examined in the next part.

### Contributing factors to the evaluation of the ongoing servicing expense of a 36-port charging station installation

**Fig. 6:**
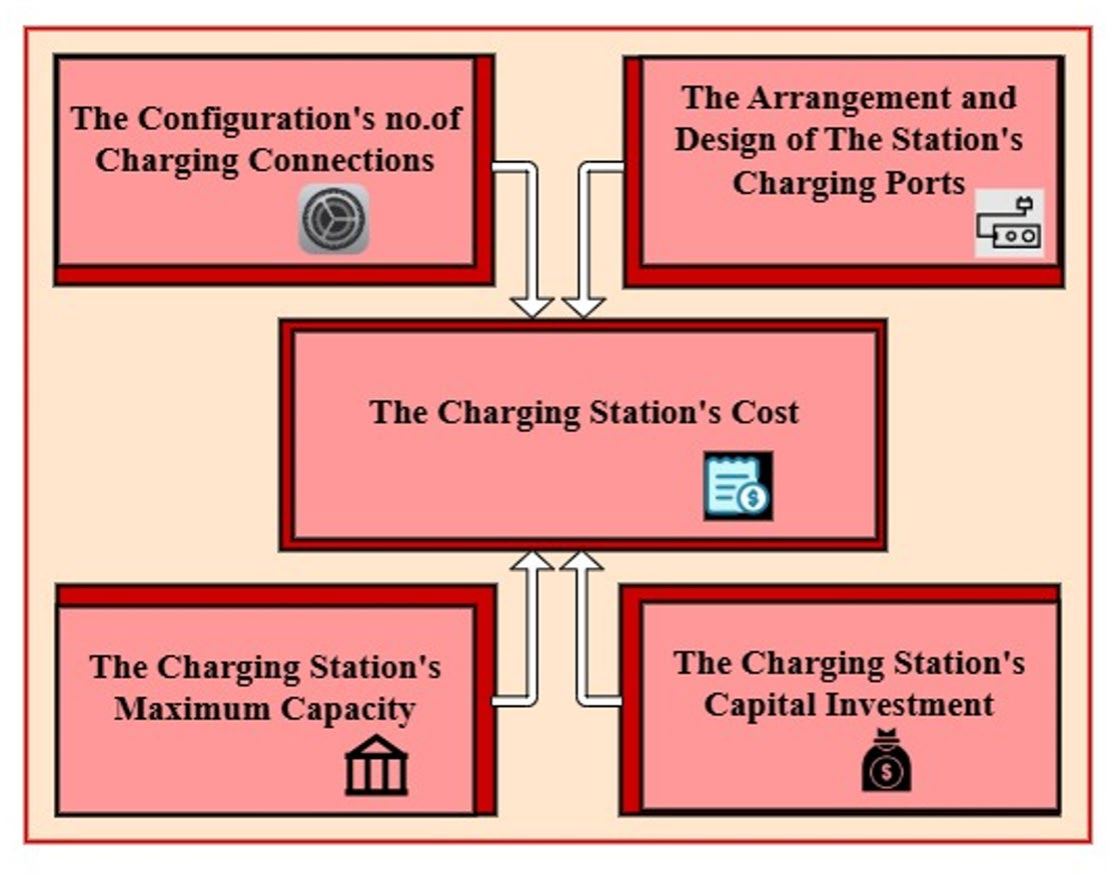
Elements that affect the charging station’s cost.

Examined were the cost elements as depicted in Fig. 6, the charging station is used to assess the cost function of capacitated stations with 36 ports. This study looked at both identical and non-identical system combinations, computing port failure rates for each type of batch port and then estimating system reliability depending on these failure rate numbers. With individual batch estimates computed using Eq. (9) to (10), cost functions were created in^33^ for each identical and non-identical charging systems.

Every port’s unique voltage

9 $$\mathop V\nolimits_{Loss}^{ind} = \frac{Total \, voltage}{{\max \, voltage}}$$

The charging station’s entire installation cost

10 $$C.F = \mathop N\nolimits_{{port + \alpha \, \mathop V\nolimits_{M.C}^{ind} }}$$

where N_port =_ Number of charging ports in charging station.

α = current rating factor of the port.

A. Comparison of healthy ports’ maintenance costs with those of failure.

A cost comparison between the proposed 36-ported charging station in both identical and non-identical configurations has been presented, taking into account failure rates and healthy port status. The maintenance costs were evaluated specifically for the 16-ported identical and 20-ported non-identical structural configurations. Per-unit (p.u.) normalization of cost and reliability metrics is adopted for fair comparison among various charging port configurations. Regarding maintenance cost, the base considered is the standard installation cost of the first charging port, and all costs thereafter are normalized in relation to that amount. To ease on reliability, the p.u. base is based on 10^6 operational hours in accordance with MIL-HDBK-217F standard with success and failure probabilities put at variance to such benchmark using normalisation ratios. Table 5 displays the comparative maintenance costs between these 16-ported and 20-ported configurations.

**Table 5:** Comparing the suggested 36-port charging station layout’s maintenance expenses.

16 & 20-Ported Batch System Combination	Batches	No. of Ports/Batch	Failure Voltage-based Maintenance Cost (F. V_MC_)	Stable Voltage-based Maintenance Cost (S. V_MC_)
4	16-ported batch system			
	B-1	4	24.5	24.5
	B-2	4	25.5	24.5
	B-3	4	26.5	24.5
	B-4	4	26.5	24.5
6	20-ported batch system			
	B-1	1	24.2	24.8
	B-2	2	24.7	25.3
	B-3	3	24.87	26.13
	B-4	4	24.5	27.5
	B-5	5	28.6	24.4
	B-6	5	28.6	24.4

The suggested charging station setup’s maintenance cost was assessed in per-unit (p.u.) terms. Table 5 shows that the 16-ported identical configuration maintains consistent costs across all batches in the port arrangement, at **26.5 p.u** This configuration provides voltage stability at **24.5 p.u.** while maintenance costs remain at **26.5 p.u.** Table 5 also reveals that the 20-ported non-identical configuration has variable maintenance costs depending on the specific ports integrated into the charging station structure. Analysis shows that charging services for this configuration can be maintained with costs ranging between **28.6** and **24.2 p.u.** The data indicates that the 16-ported identical configuration offers better performance regarding maintenance costs compared to the 20-ported non-identical configuration. Overall, the combined 36-ported identical and non-identical charging station can deliver EV charging capabilities with a maximum acceptable maintenance cost of **28.6 p.u.** Voltage stability assessments were conducted based on port failure rates, and a comparison was presented to enhance understanding of the proposed configuration.

It can be determined that while configurations^34^ utilize 13 ports, they incur significantly higher maintenance costs. Despite the proposed configuration incorporating more ports in the station, it delivers superior reliability alongside more economical maintenance expenses.

A dependable porting arrangement ensures the voltage stability of the EV charging station.

**Fig. 7:**
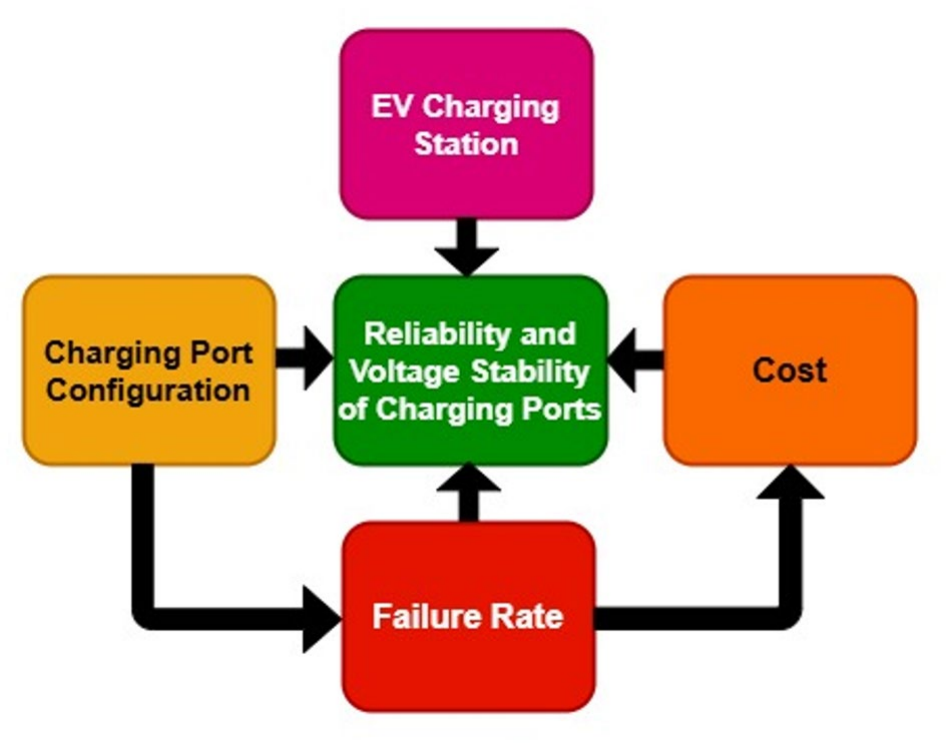
Criteria of voltage stability and dependability.

Figure 7 shows the procedure of evaluating voltage stability in layouts of EV charging stations. Many EVs congregating at charging stations can cause problems such as voltage instability, unreliability, and higher maintenance requirements. Given these difficulties, the suggested charging station has been assessed for voltage stability, dependability, and maintenance needs. The proposed station’s maintenance expenses are first calculated to start the evaluation. Examining the consistency of voltage stability guarantees more dependable charging services. Installing dependable charging stations can greatly lower running costs connected to supporting EV vehicles. Ensuring safe and dependable functioning of EV charging stations depends on voltage stability measurement. Regular voltage stability study improves charging station performance and helps to avoid instability problems. Power distribution centers with charging station systems have shown consistent performance on the IEEE-13 bus test system^35^. We added clarification that our methodology focuses on port-level reliability and maintenance-cost optimization, validated on the IEEE-13 bus system, distinguishing it from prior GA/PSO/PPF studies that focus on station siting, sizing, and scheduling.

The index of voltage stability quantifies the capacity of the power system to keep steady voltage levels on every single bus following a disturbance. The formula indicates that several variables-including real power demand, reactive power demand, line reactance, bus voltage, and the number of EV charging ports influence voltage stability index (VSI). As EV charging ports increase, both real and reactive power demands rise correspondingly. This phenomenon may reduce bus voltage, making the power infrastructure is more susceptible to voltage instability. Line reactance significantly influences, with increased reactance making the system is more susceptible to fluctuations in voltage. Bus voltage is a critical component in stability calculations—lower bus voltage increases the system’s vulnerability to instability. By thoroughly understanding these VSI influencing factors, measures can be implemented to enhance power system voltage stability and reduce voltage instability risks.

**Fig. 8:**
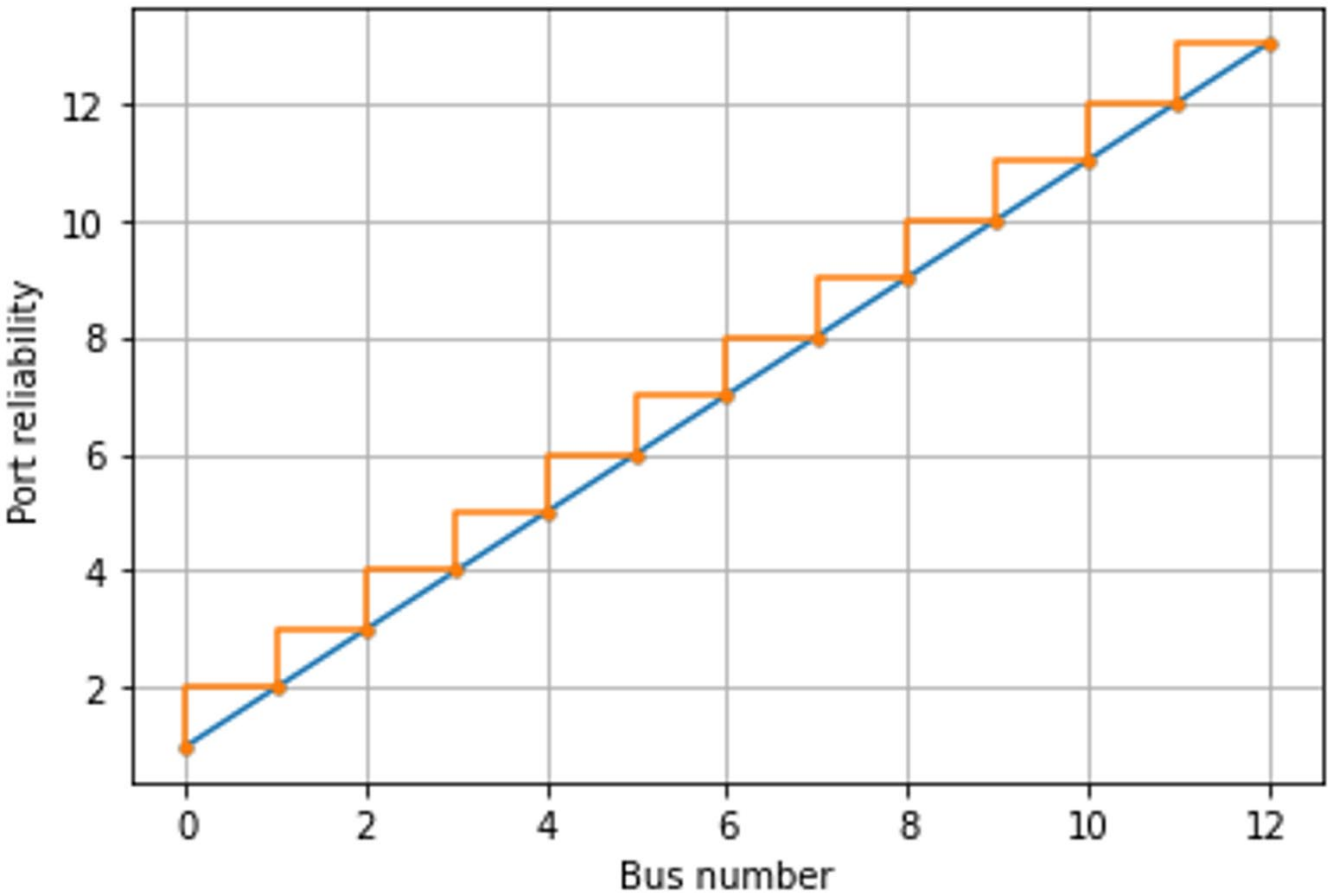
Study of voltage stability in IEEE 13-bus system to assess the EV charging station’s port dependability.

Additional factors can affect the voltage stability equation. This study examines four key aspects of EV charging ports: location, type, control method, and operational conditions within the power system. Considering all these variables allows the development of a more accurate IEEE 13 bus system’s voltage stability equation for EV charging ports. This approach has benefits such scalability for examining EV charging impacts on various-sized power systems and flexibility for exploring alternative charging scenarios. A useful instrument for examining electric car charging effects on the power grid, the EV charging port-equipped IEEE 13 bus system. This approach allows for assessing the impact of charging EVs on power supply voltage stability. Distribution system for combined identical and non-identical batched charging ports shows voltage stability reliability as shown in Fig. 8. With the suggested 36-ported charging method, failure rate and voltage stability dependability were estimated; these findings are shown in Fig. 8.

The voltage stability reliability likelihood has been assessed depending regarding the capacity and port of the proposed setup. Visually, Fig. 8 shows the supporting findings for voltage stability. Here, the blue line shows the reliability probability of each port in the 36-port setup. The orange staircase line indicates binomial probability intersections, reflecting port repeatability in the IEEE 13-bus system. The data indicates suggested 36-ported charging station may offer charging capabilities with a 22% failure probability, as shown in Fig. 8. Using binomial distribution function statistical analysis and port repeatability logic, the proposed 36-ported charging station’s dependability probability was assessed. Individual port reliability characteristics are displayed in Fig. 8. According to the repeatability logic, the system can provide charging facilities with a repeatability factor of 2. The actual 36-port probability was applied to only 13 ports due to the repeatability factor of 2 and the requirement that the first port’s initial trial remain constant. In Fig. 8, orange staircase intersection points represent each port’s reliability. For clarity, this is depicted pictorially, with each concentrating circle indicating the suitable port count for the suggested 36-ported system. The IEEE 13-bus system and 36-port setup serve as proof-of-concept, with scalability to larger networks and varied station sizes left for future work.

In the proposed 36-ported identical and non-identical configuration, two ports maintain healthy operation alongside the 13-bus system. Consequently, the failure rate reliability assessment was applied to 13 ports, accounting for the availability of 2 healthy ports in the configuration. Based on port failure rates, voltage stability reliability was evaluated for the 13-bus system. As shown in Fig. 8, each port’s reliability intersects with the 13-bus linear failure rate possibility. The first port demonstrates a minimum reliability probability of 1.0 per 10^6^ h, while the 15th port shows a maximum reliability probability of 14.2 per 10^6^ h.

## Discussion

The majority of the work on planning and reliability study for EVCS focuses either on optimal placement/ sizing of charging stations^10,11,23^ or economic cost analysis without detailed reliability assessment^7,8,12^. Although such techniques enhance distribution productivity, they fail to systematically evaluate the reliability of each charging port in various assembles. The proposed 36-port configuration, with 16 identical and 20 non-identical ports, offers the following unique advantages,

- Evaluates failure rate and reliability probability of each charging port using MIL-HDBK-217F and MIL-HDBK-338B guidelines, which most prior works did not incorporate.
- Provides a comprehensive comparison of identical vs non-identical port structures in terms of failure rate, success rate, maintenance cost, and voltage stability (Tables 3, 4 and 5, Figs. 4, 5, 6, 7 and 8).
- Demonstrates that the identical-port structure achieves a failure rate of 0.14 per 10^6 h with stable maintenance costs (24.5–26.5 p.u.), outperforming previous studies such as ^34^, which reported higher maintenance costs despite fewer ports.
- Validates voltage stability of the proposed configuration on the IEEE-13 bus system, whereas most related works (e.g.,^4,17,18^) restrict analysis to small-scale test systems or omit port-level reliability considerations.

Thus, the results establish that the proposed 36-port design offers lower failure rates, superior reliability, and reduced maintenance costs compared to state-of-the-art methods, demonstrating both novelty and practical significance.

## Limitations

### Assumption of standardized reliability data

The estimate rate of failure is according to MIL-HDBK-217F and MIL-HDBK-338B. While these models are generally accepted, they are not necessarily representative for field level uncertainties like product variance, local climatological conditions and user behaviour.

• Real-world field validation under civil environmental factors (temperature fluctuations, humidity, dust accumulation, and irregular user handling) remains an important future step.

• Our methodology is extendable to incorporate empirical field data once such measurements become available, which would further enhance the accuracy of the reliability predictions.

### Modeling of the load and user behavior simplified

The consideration is made under random plugin condition and average charging behavior. Yet in reality, charging patterns could be quite diverse according to time of use tariff, traffic jam density or customer habit, which may challenge the reliability prediction.

### Limited grid interaction analysis

Meanwhile, the voltage stability was verified through IEEE 13-bus system under regulated conditions. Full scale assimilation into real urban distribution networks could bring other complications, for example, power harmonics, renewables intermittency and/or demand-response dynamics involvement.

### Economic factors not exhaustively modeled

The cost model for maintenance accounted port level voltage stability and failures rates. A broader market related aspects as well as framework ecological effects (land cost, electricity price policies and the V2G integration incentives among others) were not included within current project frame.

### Scalability considerations

The design concept was implemented for the 36-ports. Although this approach is scalable, its computational effectiveness and trustworthiness should be further verified for the extremely large-scale EV charging hubs.

## Conclusion

In this paper, we divided the study by analyzing the reliability of EV charging stations for two case series with similar capacity in all stations (identical ports) and different in each station (non-identical ports). A practical design of a 36-port charging station, including the combination of 16 identical ports and 20 non-identical ports, is developed for actual application on a distribution system with a capacity from 50 to 350 kW. The reliability evaluations were performed by sequentialization for component failure evaluation according to MIL-HDBK-217F. It has been shown that the failure probability in disimilar port configurations is higher than that in similar ones (eg, 2% failure rate; 0.5 per 10^6 h). The maintenance costs, in terms of a port failure rate, are also less for identical-configuration ports **(24.5/26.5 p.u.)** than they are for non-identical configuration **(25/26 p.u.)**. The mixed 36-port arrangement presented achieves the trade-off among availability, maintenance, and performance required, providing high EV charging service ratio efficiently. Such results validate the proposal of such a configuration when implementing V2G-enabled operation, with economic and reliability benefits over conventional identical or non-identical port layouts.

## Future scope

Both identical and non-identical configurations can be explored for a more reliable EV port setup. Evaluating and comparing these configurations can help analyze failure rates, assess reliability, and estimate costs for procuring suitable products. In addition, future work will integrate region-specific cost databases (equipment cost, land value, and labor cost) to provide absolute monetary estimates for maintenance and investment, thereby enhancing the decision-making relevance of the proposed framework. The study can be expanded to larger IEEE test systems and real-world distribution networks to evaluate scalability and performance under practical urban conditions.

## Data Availability

Availability of data and materials, the dataset used and analyzed during the current study is not publicly available as per institutional norms. But the data can be made available by the corresponding author upon reasonable request.

## Abbreviations

EV: Electric vehicle
EVCS: Electric vehicle charging station
V2G: Vehicle-to-grid
VSI: Voltage stability index
SOC: State of charge
DG: Distributed generation
PEV: Plug-in electric vehicle
P.U.: Per unit
DSVS: Dynamic system voltage stability
CINPS: Combination of identical and non-identical port systems
